# Early detection of ocular abnormalities in a Chinese multicentre neonatal eye screening programme—1‐year result

**DOI:** 10.1111/aos.14586

**Published:** 2020-09-15

**Authors:** Ping Fei, Zijiang Liu, Liying He, Na Li, Lihua Xu, Meiju Zhang, Yu Zhou, Fuxin Li, Hong Wang, Qi Zhang, Qiujing Huang, Yi'an Li, Shuangshuang Chen, Wei Guo, Yun Li, Ya Liu, Jun Lu, Ying Wang, Xiuyu Zhu, Lei Wang, Yanhong Wang, Jianying Xian, Yu Xu, Xunda Ji, Tingyi Liang, Jianing Ren, Xi Zhang, Jing Li, Peiquan Zhao

**Affiliations:** ^1^ Department of Ophthalmology Xinhua Hospital Affiliated to Shanghai Jiaotong University School of Medicine Shanghai China; ^2^ Department of Ophthalmology Urumqi Maternal and Child Health Hospital Xinjiang China; ^3^ Department of Ophthalmology Chongqing Health Center for Women and Children Chongqing China; ^4^ Department of Ophthalmology Kunming Maternity and Child Care Hospital Kunming city China; ^5^ Department of Ophthalmology Dezhou Women's and Children's Hospital Shandong China; ^6^ Department of Ophthalmology Women and Children’s Health Care Hospital of Linyi Shandong China; ^7^ Department of Ophthalmology Maternity and Child Health Care of Guangxi Zhuang Autonomous Region Giangxi China; ^8^ Department of Ophthalmology Jilin Women and Children Health Hospital Jilin China; ^9^ Department of Ophthalmology Maanshan Wemen and children's Hospital Anhui China; ^10^ Clinical Research Unit Xinhua Hospital, Affiliated to Shanghai Jiaotong University School of Medicine Shanghai China

**Keywords:** eye screening, neonatal, retinal hemorrhage, retinoblastoma

## Abstract

**Purpose:**

Early detection of ocular abnormalities in newborns is essential for timely diagnosis and treatment. This study aimed to assess the 1‐year result of a multicentre prospective neonatal eye examination programme with wide‐field digital imaging system in China.

**Methods:**

A multicentre collaborative prospective study group for neonatal eye screening was established in nine hospitals, including eight Maternal and Children's Hospitals, and one general hospital across China from July 2016 to June 2017. Ocular examinations were performed on newborns within 28 days after birth using a wide‐field digital imaging system. Data were reviewed and analysed. The primary outcome was the prevalence of ocular abnormalities in neonates.

**Results:**

We detected 13 514 (20.91%) abnormal cases in 64 632 newborns. The most frequent abnormality was retinal haemorrhage (RH; 11.83%). Most of mild RH resolved spontaneously. Among those who were beyond retinopathy of prematurity (ROP) screening criteria of China (gestational age ≥32 w and birthweight ≥2000 g), the total number of neonates with ocular abnormality was 12 218/62 799(19.45%). 59.44% of neonatal ocular abnormalities detected (accounting for 11.56% of all the screened population) needed further interference or observation. Among them, 258 patients (0.41% of all the screened population) needed immediate or timely intervention, including congenital cataract, retinal detachment, retinoblastoma and other ocular abnormalities. One thousand and ninety‐eight patients (1.75% of all the screened neonates) should be followed up closely and needed further diagnosis or intervention if necessary, such as ROP or ROP‐like retinopathy, familial exudative vitreoretinopathy and persistent hyperplasia of primary vitreous. Five thousand nine hundred and six patients (9.4%) with minor clinical significance needed short‐term follow‐up.

**Conclusions:**

This prospective multicentre study of newborn ocular examination showed a relatively high prevalence of ocular abnormalities. There are a relatively high percentage of congenital eye pathology that required further referral and treatment in those neonates who were not screened routinely. According to the benefits and risks associated with neonatal eye examinations, neonatal ocular screening programme can detect ocular abnormalities at the very early stage and may play a positive role in promoting paediatric eye health.

## Introduction

Because visual development commences soon after birth, screening for ophthalmic disorders in the newborn is of paramount importance to identify amblyogenic factors. The American Academy of Pediatrics recommends red reflex testing (RRT) shortly after birth (AMERICAN ACADEMY OF PEDIATRICS, 2008). However, some ocular disorders cannot be detected by RRT (Eventov‐Friedman et al., 2010; Sun et al., 2016; Cagini, 2017; Ludwig et al., 2018). The importance of ophthalmologic evaluation in the newborn was underscored by Wasilewski and coworkers (Wasilewski et al., 2002) in 2002, who reported that 3.75% of 667 newborns showed some ocular abnormalities, of which 56% was not identified by the infant's paediatrician. The most commonly observed ophthalmic disorder in the study was corneal opacity.

Newborn eye screening is an emerging concept for early detection and intervention of many eye diseases that are present at birth since the commencing of the wide‐field digital retinal imaging (WFDRI). several studies reported the incidence of eye abnormalities (Vinekar et al., 2015; Callaway et al., 2016; Goyal et al., 2017; Li et al., 2017; Ma et al., 2018; Simkin et al., 2019), including retinal haemorrhages (RHs) in healthy full‐term neonates. The initial outcomes of the newborn eye screening programme suggested that early screening was valuable in making an early diagnosis of visual abnormalities. The studies also showed that the incidence of ocular abnormalities was higher than expected. However, most of them were single centre studies, and the incidences of neonatal ocular abnormalities varied from 4.7% to 24.40% (Vinekar et al., 2015; Callaway et al., 2016; Goyal et al., 2017; Li et al., 2017; Ma et al., 2018; Simkin et al., 2019). In this study, we aimed to report the 1‐year results of a large multicentre prospective neonatal eye examination programme with wide‐field digital imaging system and the percentage of the ocular abnormalities which needed intervention or follow‐up after early detection.

## Methods

### Study design

A multicentre collaborative prospective study group for neonatal eye screening was established within nine hospitals, including eight Maternal and Children's Hospitals, and one general hospital. These hospitals are all qualified for neonatal eye screening before joining this study group. They are located in seven different provinces of China. Collectively, they covered North, South, East, West and Center China. Xinhua Hospital affiliated to Shanghai Jiao Tong University School of Medicine coordinated this study. Data collected from all participating hospitals were kept and analysed in Xinhua Hospital.

The study protocol was approved by the Ethics Committees of Xinhua Hospital, adopted and approved by the ethics committees of other participating hospitals. Informed consent was obtained from all subjects participating in this study. This study was registered on Clinialtrial.org (NCT02851251).

### Patients and data collection

The source population was infants born at the above eight Maternal and Children's Hospitals. The study sample represented subjects whose parents and paediatricians consented to the study and the infant completed the screening procedure.

Inclusion criteria were all live neonates at all these eight Maternal and Children's Hospitals whose parents had signed the consent form from July 2016 to June 2017. Premature infants as defined by Chinese Ophthalmological Society were also included [infants born <32 weeks gestational age (GA) or <2000 g birthweight].

Exclusion criteria were infants whose images or medical charts were not available for review, patients with infectious conjunctivitis, and patients deemed too unstable for examinations by their attending paediatrician.

### Photography protocol

Red reflex of each eye was recorded with the ophthalmoscope before photography. RetCam III (Natus Medical Incorporated, San Carlos, CA, USA) with 130 degree lens was used to obtain wide‐angle images of all infants enrolled in this study. The standard external examination, anterior segment examination and fundus examination by RetCam III were performed within 28 days after birth by well‐trained ophthalmologists with the assistance of nurses (holding the baby) at each centre. We took five fundus photographs (nerve up, down, left, right and centre) for each patient, and also photographs of external eye and anterior segment if there were any abnormalities. Pupil was dilated before examination using tropicamide phenylephrine eye drops for 3–4 times with a 10‐min interval. Topical anaesthetic was instilled in each eye prior to imaging. A sterile speculum was used during screening to allow adequate ocular exposure for anterior segment and retinal imaging. Liquid gel was used as a coupling agent between the RetCam lens and cornea.

All images and data including gender, GA, age at screening, birth modality, birthweight and age of mother when available were collected for analysis. Images of eyes with abnormal presentation were sent to the referral centre for further review. Infants with RH and other ocular abnormalities with potential visual or systemic impact were referred for further follow‐up or treatment.

### Quality control

Quality control was focused on completeness and accuracy of the examinations. Staffs committed to the study were trained to ensure the application of the same criteria for examinations and diagnosis. Data from each centre were reported to the coordinator every month. Reported eye abnormalities were double‐checked by the coordinators in the reading centre of Xinhua Hospital.

### Outcomes

The primary outcome of this study was the prevalence of ocular abnormalities in neonates. The secondary outcome was the percentage of ocular abnormalities which needed intervention or follow‐up.

### Statistical analysis

Statistical analysis was performed using SAS software version 9.4 (SAS Institute Inc., Cary, NC, USA). Continuous variables were summarized as the median with 25th and 75th percentiles, or the mean ± standard deviation (SD), depending on whether their distributions were or were not highly skewed. A value of p < 0.05 was considered statistically significant in all tests if applied.

## Results

### Demographics and characteristics of screened population

During the 1‐year study period, 64 632 newborns participated in the screening. The average birthweight was 3314.84 ± 541.66 g. The average GA was 39 (38, 40) weeks. The average age at screening was 4 days (1, 7). The male‐to‐female ratio was 1.18:1. We classified the screened population into two groups. Group 1 included 1833 (2.84%) infants who were born prematurely as defined by Chinese retinopathy of prematurity (ROP) guideline. The rest of the babies (62 799) were classified as Group 2 (Table 1).

**Table 1:** Demographics and characteristics of neonates enrolled in multicentre universal newborn eye screening.

	Total neonates	Group 1*	Group 2*
Total number (%)	64 632 (100)	1833 (2.84)	62 799 (97.16)
Gestational age (weeks)	39 (38, 40)^†^	32 (31, 34)^†^	39 (38, 40)^†^
Age at screening (days)	4 (1, 7)^†^	15 (7, 23)^†^	4 (1, 7)^†^
Age of mothers (years old)	29 (27, 32)†	29 (27, 32)^†^	30 (27, 33.5)^†^
Birthweight (grams, SD)	3314.84 ± 541.66	1678.56 ± 383.00	3362.34 ± 466.56
Gender			
Male (%)	34 939 (54.06)	949 (51.77)	33 990 (54.13)
Female (%)	29 693 (45.94)	884 (48.23)	28 809 (45.87)

* Group 1 included those neonates who were premature [gestational age (GA) <32 w] or low birthweight (<2000 g; Chinese retinopathy of prematurity screening criteria). Group 2 included the remaining neonates beyond screening criteria (GA ≥32 w) and birthweight (≥2000 g).

^†^ Variables were summarized as the median with 25th and 75th percentiles.

### Ocular abnormalities detected by the screening programme

**Fig. 1:**
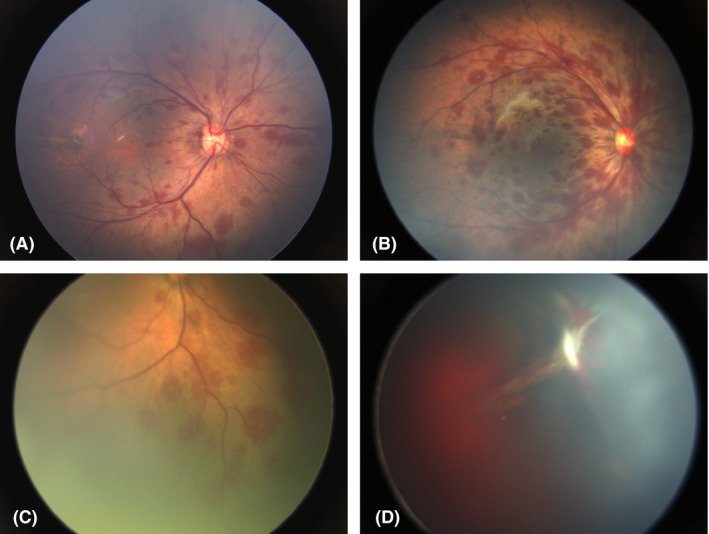
Retinal haemorrhage (RH) found in the newborn eyes. (A) Mild RH; (B) Moderate RH; (C) Retinal haemorrhage (RH) with white centre (Roth's spot); (D) Severe RH with vitreous haemorrhage and persistent hyperplasia of primary vitreous (PHPV).

The total number of infants with ocular abnormality was 13 514, which accounted for 20.91% of all the screened neonates. The most common abnormality was RH, which was detected in 7648 (11.83%) cases, including 4553 (7.04%) cases involved bilaterally (Fig. 1). Three hundred and forty‐eight (0.54%) neonates had foveal haemorrhage, which was potentially vision threatening. None of the screened neonates had any significant adverse events during or after the examination. The abnormalities were classified into three types: external eye and systemic abnormalities, abnormalities of the anterior segment and abnormalities of the posterior segment. Red reflex detected 94.8% (492 out of 519 cases) of the anterior segment abnormalities. However, only 3.97% (614 cases out of 15 456) of the posterior segment abnormalities, such as persistent hyperplasia of primary vitreous (PHPV), severe RH and choroidal coloboma, were detected correctly by the red reflex test.

In Group 1, 1296/1833 (70.70%) babies had ocular abnormalities, including 57.01% with immature retina. Retinopathy of prematurity (ROP) was observed in 165 infants (9.00%). Other abnormalities were similar compared with the total screened population (Table 2).

**Table 2:** Abnormal ocular findings in premature or low birthweight infants within ROP screening criteria (Group 1).

Ocular abnormalities	Patients No. of ocular abnormalities (%*)
External eye and systemic abnormalities	
Neonatal fundal jaundice	2 (0.11)
Abnormalities of anterior segment	
Congenital cataract	6 (0.33)
Corneal oedema	3 (0.16)
Subconjunctival haemorrhage	1 (0.05)
Leucoma	1 (0.05)
Abnormalities of posterior segment	
Immature retina	1045 (57.01)
ROP	165 (9.00)
Total retinal haemorrhage	70 (3.82)
Retinal venous tortuosity	50 (2.73)
PHPV	24 (1.31)
Pigment deposition	18 (0.98)
Peripheral retinal white lesion	14 (0.76)
Retinal exudates	10 (0.55)
Depigmentation	8 (0.44)
Vitreous opacity	6 (0.33)
Optic disc abnormalities	5 (0.27)
Optic dysplasia	4 (0.22)
Retinitis	3 (0.16)
FEVR	2 (0.11)
Choroidal coloboma	2 (0.11)
Maculopathy	1 (0.05)
Hypopigmented fundus	1 (0.05)
Optic atrophy	1 (0.05)
Total No. of ocular abnormalities	1296 (70.70)

FEVR = familial exudative vitreoretinopathy, MGS = Morning glory syndrome, PHPV = persistent hyperplasia of primary vitreous, RNFL = myelinated retinal nerve fibre Layer, ROP =^ ^retinopathy of prematurity.

* Per cent of abnormalities accounting for total ocular abnormalities.

**Fig. 2:**
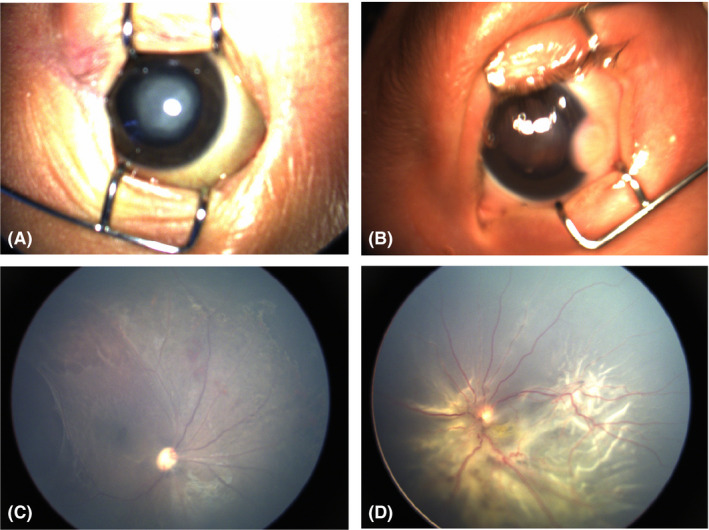
Ocular abnormalities detected in the neonates needed immediate or timely intervention. (A) Congenital cataract; (B) Corneal dermoid; (C) Retinitis; (D) Retinal detachment.

**Fig. 3:**
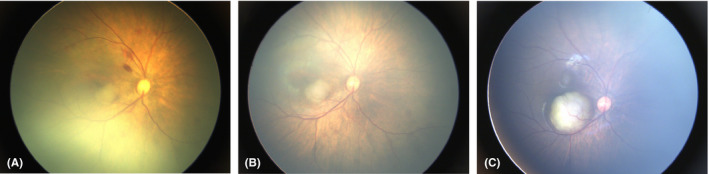
Retinoblastoma. (A) Macular mass (suspected retinoblastoma) detected in a male neonate during eye screening. (B) The lesion had slightly enlarged 10 days later. The parents refused further immediate treatment and lost follow‐up after the screening. Three months later, he revisited the clinic and the fundus examination revealed the mass was enlarged dramatically. The parent agreed to have further treatment finally and the patient was treated appropriately.

**Fig. 4:**
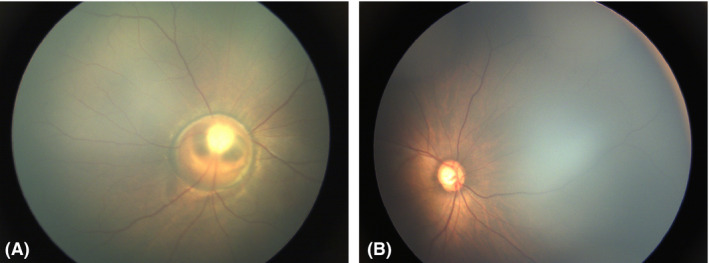
Optic abnormalities. (A) Optic coloboma; (B) Enlarged cup/disc ratio in neonates.

In Group 2, the total number of neonates with ocular abnormalities was 12 218 (19.45%), which was similar but slightly lower than the percentage among total population 13 514/64 632 (20.91%). All these abnormalities in Group 2 were classified into different categories according to whether they needed intervention or follow‐up. Since most of mild and moderate RHs could be absorbed spontaneously (Emerson et al., 2001), only those involving macula were included in this analysis (Table 3). All these three types accounted for 59.44% of total ocular abnormalities detected and 11.56% of all the screened neonates. Two hundred and fifty‐eight patients (0.41% of all the screened population) needed immediate or timely intervention, including those with severe consequences such as congenital cataract, retinal detachment and retinoblastoma (Fig. 2, 3). One thousand and ninety‐eight patients (1.75% of Group 2) should be followed up closely and need further diagnosis or intervention if necessary, such as ROP or ROP‐like retinopathy, familial exudative vitreoretinopathy (FEVR) and PHPV. Some ocular findings were indicative of systematic abnormalities. The early identification of these untreatable ocular disorders such as congenital anomalies of the disc can also prompt further investigations (Fig. 4), including genetic and systemic investigation. These patients needed further investigation or intervention. The remaining 5096 patients (9.4%) with ocular abnormalities might have minor clinical significance, but still needed short‐term follow‐up, such as immature retina, peripheral retinal white lesions and retinal exudates.

**Table 3:** Ocular abnormalities* according to different intervention/follow‐up among neonates beyond screening criteria (Group 2).

Immediate/ timely Intervention		Intervention/close or long‐term follow‐up		Short‐term follow‐up	
Ocular abnormalities	No. of patients (%^†^)	Ocular abnormalities	No. of patients (%^†^)	Ocular abnormalities	No. of patients (%^†^)
Congenital cataract	156 (1.28)	FEVR	283 (2.32)	Peripheral retinal white lesion	1321 (10.81)
Conjunctivitis	63 (0.52)	ROP (like) retinopathy	241 (1.97)	Retinal exudates	1236 (10.12)
Corneal Oedema	12 (0.10)	Maculopathy	141 (1.15)	Immature retina	1149 (9.40)
Retinitis	11 (0.09)	PHPV	115 (0.94)	Pigment deposition	692 (5.66)
Retinoblastoma	5 (0.04)	Choroidal coloboma	97 (0.79)	Depigmentation	434 (3.55)
Neonatal dacryocystitis	4 (0.03)	Vitreous opacity	64 (0.52)	Foveal Haemorrhage	348 (2.85)
Iris neovascularization	2 (0.02)	Optic dysplasia	64 (0.52)	Subconjunctival haemorrhage	265 (2.17)
Leucocoria	2 (0.02)	Enlarged optic cup	51 (0.42)	Retinal venous tortuosity	170 (1.39)
Exophthalmos	1 (0.01)	Strabismus	18 (0.15)	White without pressure	117 (0.96）
Corneal dermoid	1 (0.01)	Optic coloboma	5 (0.04)	Neonatal fundal jaundice	107 (8.76)
Retinal detachment	1 (0.01)	Angioma of eyelid	5 (0.04)	Hypopigmented fundus	35 (2.86)
		Optic nerve head capillary haemangioma	4 (0.03)	Albinotic fundus	15 (1.23)
		Nystagmus	4 (0.03)	Abnormal shape of optic disc	11 (0.09)
		Persistent pupillary membranes	3 (0.02)	Iris synechia	4 (0.03)
		Optic atrophy	2 (0.02)	MRNF	2 (0.02)
		MGS	1 (0.01)		
Total	258 (2.11)		1098 (8.99)		5906 (48.34)
Per cent of abnormalities accounting for total screened population (%)	0.41		1.75		9.40

FEVR = familial exudative vitreoretinopathy, MGS = morning glory syndrome, MRNF = myelinated retinal nerve fiber layer, PHPV = persistent hyperplasia of primary vitreous, ROP = retinopathy of prematurity.

* Only retinal haemorrhage involving macula was listed in the table.

^†^ Per cent of abnormalities accounting for total ocular abnormalities.

## Discussion

This study presented the 1‐year result of multicentre newborn eye screening in China. We compared the results reported by other groups (Table 4). Our results were largely comparable with previously reports. Most of previous studies have been based in single centre. To our knowledge, this is the first prospective multicentre neonatal eye screening reported and presented both basic information and ocular findings. Our study included hospitals from different parts of China, and each hospital had a neonatal screening percentage of over 75%. With large number of babies enrolled in, the data obtained here were representative of neonatal eye abnormalities across China.

**Table 4:** Present study in comparison to the other major studies of similar kind.

Publications	Country	Single centre/multicentre	Screened population	Screened number	Method	Timing	Ocular pathology	RHs	Other abnormal findings
Li et al. (2013)	South China	Single centre	BW >2500 g and 37 weeks GA	3573	Flash light, Retinoscope, S/L WFDRI	<42 days after birth	24.40%	21.50%	Sub conjunctival haemorrhage (1.4%), microphthalmos, congenital cataract, corneal opacity, Hamartoma/Rb
Vinekar et al. (2015)	South India	Single centre	Term and BW 42 kg	1021	WFDRI	72 hr after birth	4.70%	2.4%	Ridge‐like ROP (0.9%), retinal dysplasia, PFVS, RB, congenital cataract
Li et al. (2017)	South China	Single centre	Full‐term neonates	15 284	WFDRI	Within 7 days after birth	21%	19%	Media opacity, optic nerve abnormalities (coloboma), cataract, RB
Goyal et al. (2017)	Eastern India	Single centre	Healthy neonates any BW and GA	1152	WFDRI	<28 days after birth	14.90%	13.20%	Congenital glaucoma, ridge‐like ROP, cystic fovea, uveal coloboma
Callaway et al. (2016)	US	Single centre	All infants who were 37 weeks postmenstrual age or older and were deemed stable	202	WFDRI	131 infants <48 hr; 71 infants ≥48 hr	20.30%	20.30%	Fundus haemorrhages
Simkin et al. (2019)	New Zealand	Two centre	Infants excluding GA <3 weeks or BW <1250 g	350	WFDRI	Not specified	15.90%	14.50%	Congenital cataract and optic nerve hypoplasia
Our study	China	Multicentre	Healthy neonates any BW and GA	64 632	WFDRI	<28 days after birth	20.91%	11.83%	Abnormalities of anterior segment, posterior segment, ocular appendix and others (Table 2)

BW = birthweight, GA = gestational age, RB = retinoblastoma, RH = retinal haemorrhage, ROP retinopathy of prematurity, WFDRI = wide‐field digital retinal imaging.

Retinal haemorrhage (RH) was the single most frequently seen ocular anomaly among newborn babies. Most of the mild and moderate RH can be absorbed quickly without leaving a trace and causing no discernible effect on visual development. However, prolonged severe RH and macular foveal haemorrhage may leave a long‐term impact on vision and ultimately lead to deprivational amblyopia. Macular foveal haemorrhage accounted for 0.54% in our study, which was similar to Vinekar's study (0.34%, FH/RH/Total population, 4/153/1152) (Goyal et al., 2017). While in Li's study, 2.8% of the full‐term neonates had foveal haemorrhage (Li et al., 2017). The variations may be due to the difference of screening timing, population and delivery methods (Table 4).

There are few literatures that have discussed the percentage of ocular abnormalities requiring further interference or follow‐up. In Vinekar's study, medical or surgical intervention of any form was required in nine babies, which accounted for 18.8% of abnormalities detected and 0.9% of all babies screened. Our screening programme revealed 11.56% of all the screened neonates in Group 2 needed intervention or follow‐up, which accounted for 59.44% of the total ocular abnormalities detected. Among them, 2.16% of the screened population required timely intervention or further investigation and long‐term follow‐up. Some ocular abnormalities needed urgent treatment, such as congenital cataract, corneal oedema, retinal detachment and retinoblastoma. Our data showed higher percentage which needed intervention than Vinekar's. It is possibly due to the difference of screened population and the setting of the multicentre study.

We identified five cases (0.01%) of confirmed retinoblastoma without family history during the screening. All were at very early stages of RB and received timely treatment. In the United States, the mean age of children at the diagnosis of retinoblastoma was 25 months for unilaterally affected ones, and 15 months for the bilaterally affected ones (Abramson et al., 2002). Only when there is a known family history and children are screened for the disease, the mean age at diagnosis is younger than 1 year. In the reported study, despite the diagnosis within 1 year, or even 6 months of life, the most common intraocular Reese‐Ellsworth group at diagnosis was group V (i.e. massive tumours involving more than half of the retina and vitreous seeding) and the most common manifesting sign or symptom was leucocoria (Abramson et al., 1985, Abramson and Servodidio, 1992). However, in our study, all the screened RBs were at the very early stages, which had very good prognosis after timely treatment. The early treatment of retinoblastoma can not only be vision preservation but also be life‐saving.

The early identification of other severe ocular disorders such as congenital anomalies of the optic disc can also prompt further investigations, including genetic and systemic investigation, such as neuro‐imaging or endocrine investigations. For example, morning glory syndrome (MGS) may have facial and central nervous system abnormalities, such as basal encephalocele and pituitary dwarfism (Caprioli and Lesser, 1983, Itakura et al., 2013; Minotto et al., 2007; Fei et al., 2013). A complete general physical examination and growth evaluation is important for early detection and treatment, resulting in benefit for these patients. Rehabilitation on functional amblyopia in organic optic nerve anomalies is essential. Occlusion therapy during the period of sensory maturation can prevent deep amblyopia in MGS patients (Loudot et al., 2007). The patients with MGS may complicate retinal detachment in the future (von Fricken and Dhungel, 1984, Yamakiri et al., 2004). Further intervention and long‐term follow‐up were essential in these patients.

A red reflex test or just an external eye examination could have detected many of the abnormalities (AMERICAN ACADEMY OF PEDIATRICS, 2008, Sun et al., 2016; Ludwig et al., 2018). However, there are many other ocular abnormalities, especially retinal pathologies could not be detected by RRT, a late detection of which could lead to worse prognosis. Wasilewski's study showed the importance of using an ophthalmologic evaluation as a routine procedure when examining newborns, which revealed that 56% of ophthalmic disorders observed at birth had not been identified by neonatologists and paediatricians using RRT (Wasilewski et al., 2002). Neonatal eye examination with wide‐field digital imaging system is prompt and efficient compared with regular ophthalmologic evaluation, which make newborn eye screening possible.

One of the most frequently encountered difficulties in doing a large scale screening of neonatal screening is the cost effectiveness. The entire cost of the screening among Group 2 neonates was estimated as Chinese Yuan (CNY) 18.84 million (US$ 2.69 million), which translated to CNY 300 (US$ 42.86) per person. The total expenses included the use of equipment, manpower, stationeries and other accessories. The inexpensive screening cost was due to low cost of manpower in China which may be variable in other countries. Excluding those which can be detected by red reflex, and mild or spontaneous recovered abnormalities of posterior segment, the screening programme detected 283 cases of FEVR, five cases of retinoblastoma, 14 cases of retinitis. A conservative estimate of 25 neonates can be saved from irreversible blindness. The minimum average yearly per capita income in China in 2018 was CNY 28 228 (US$ 4032.57). Assume that a blind individual made 1/5 of the averaged income, we calculated that the financial loss incurred due to 25 children going blind at CNY 40.55 million (US$ 5.79 million; Table 5). It is clear that newborn screening with WFDRI merits over red reflex test for all newborn within the first month of life.

**Table 5:** Estimated costs and benefits of neonatal eye screening project

Disease	① Total number of patients detected by our procedure	② Estimated average cost for treatment after early detection [CNY (US dollars)]	③ Average cost for treatment with delayed detection (CNY (US dollars))	④ Direct medical benefit (①*（③ − ②)) [CNY (US dollars)]	⑤ Estimated rate of blindness	⑥ Number of patients saved from irreversible blindness by screening (①*⑤)	⑦ Average life expectancy	⑧ Minimum average yearly per capita income [CNY (US dollars)]	⑨ Average yearly per capita income for the blind(estimated 1/5 of the average) [CNY (US dollars)]（⑧*1/5）	⑩ Indirect benefit ⑥*⑦*(⑧‐⑨) [CNY (US dollars)]	Gross benefit [CNY (US dollars)]
Familial exudative vitreoretinopathy	283	15 000 (2142.86)	50 000 (7142.86)	9 905 000 (1 415 000)	5%	14					
Retinoblastoma	5	30 000 (4285.71)	150 000 (21428.57)	600 000 (85714.29)	90%	4					
Retinitis	14	5000 (714.29)	20 000 (2857.14)	210 000 (30 000)	50%	7					
Subtotal				10 715 000 (15.0714.29)		25	70	28 228 (4032.57)	5645.6 (1128.12)	40546699.2 (5792385.6)	51261699.2 (7323099.89)

There are some limitations of this study. First, the detailed information of RH was not discussed in this paper, such as the location and severity of RHs, risk factors and the time to be absorbent. Second, the group 2 population was not completely the same compared with other countries because the ROP screening guideline varies in different countries. However, as shown in Table2 and 3, the percentage of the ocular abnormalities in Group 1 and 2 were similar except ROP and immature retina. Third, there were still some ocular abnormalities, which needed to be further investigated to make the final diagnosis, such as corneal opacity, increased cup/disc ratio, peripheral retinal white lesions and retinal exudates. These abnormalities may further deteriorate without close follow‐up and treatment. It put the emphasis on the screening of neonatal eye screening.

This prospective multicentre study of newborn ocular examination showed a relatively high prevalence of ocular abnormalities. There are a relatively high percentage of congenital eye pathology that required further referral and treatment in those neonates who were not screened routinely. According to the benefits and risks associated with neonatal eye examinations, neonatal ocular screening programme can detect ocular abnormalities at the very early stage and may play a positive role in promoting paediatric eye health.

## References

[aos14586-bib-0001] Abramson DH & Servodidio CA (1992): Retinoblastoma in the first year of life. Ophthalmic Paediatr Genet 13: 191–203. 1488219 10.3109/13816819209105167

[aos14586-bib-0002] Abramson DH , Ellsworth RM , Grumbach N & Kitchin FD (1985): Retinoblastoma: survival, age at detection and comparison 1914–1958, 1958–1983. J Pediatr Ophthalmol Strabismus 22: 246–250. 4078667 10.3928/0191-3913-19851101-11

[aos14586-bib-0003] Abramson DH , Du TT & Beaverson KL (2002): (Neonatal) retinoblastoma in the first month of life. Arch Ophthalmol 120: 738–742. 12049578 10.1001/archopht.120.6.738

[aos14586-bib-0004] AMERICAN ACADEMY OF PEDIATRICS, S. o. O., AMERICAN ASSOCIATION FOR PEDIATRIC OPHTHALMOLOGY AND STRABISMUS, AMERICAN ACADEMY OF OPHTHALMOLOGY, AMERICAN ASSOCIATION OF CERTIFIED ORTHOPTISTS (2008): Red reflex examination in neonates, infants, and children. Pediatrics 122: 1401–1404. 19047263 10.1542/peds.2008-2624

[aos14586-bib-0005] Cagini C (2017): Red reflex screening highly sensitive for anterior segment abnormalities. J Pediatr 184: 235–238. 10.1016/j.jpeds.2017.02.053 28434567

[aos14586-bib-0006] Callaway NF , Ludwig CA , Blumenkranz MS , Jones JM , Fredrick DR & Moshfeghi DM (2016): Retinal and optic nerve hemorrhages in the newborn infant: one‐year results of the Newborn Eye Screen Test Study. Ophthalmology 123: 1043–1052. 26875004 10.1016/j.ophtha.2016.01.004 PMC4918466

[aos14586-bib-0007] Caprioli J & Lesser RL (1983): Basal encephalocele and morning glory syndrome. Br J Ophthalmol 67: 349–351. 6849854 10.1136/bjo.67.6.349 PMC1040063

[aos14586-bib-0008] Emerson MV , Pieramici DJ , Stoessel KM , Berreen JP & Gariano RF (2001): Incidence and rate of disappearance of retinal hemorrhage in newborns. Ophthalmology 108: 36–39. 11150261 10.1016/s0161-6420(00)00474-7

[aos14586-bib-0009] Eventov‐Friedman S , Leiba H , Flidel‐Rimon O , Juster‐Reicher A & Shinwell ES (2010): The red reflex examination in neonates: an efficient tool for early diagnosis of congenital ocular diseases. Isr Med Assoc J 12: 259–261. 20929074

[aos14586-bib-0010] Fei P , Zhang Q , Li J & Zhao P (2013): Clinical characteristics and treatment of 22 eyes of morning glory syndrome associated with persistent hyperplastic primary vitreous. Br J Ophthalmol 97: 1262–1267. 23878133 10.1136/bjophthalmol-2013-303565 PMC3786642

[aos14586-bib-0011] von Fricken MA & Dhungel R (1984): Retinal detachment in the Morning Glory syndrome. Pathogenesis and management. Retina 4: 97–99. 6463401 10.1097/00006982-198400420-00004

[aos14586-bib-0012] Goyal P , Padhi TR , Das T et al. (2017): Outcome of universal newborn eye screening with wide‐field digital retinal image acquisition system: a pilot study. Eye 32: 67–73. 28737759 10.1038/eye.2017.129 PMC5770699

[aos14586-bib-0013] Li, LH , Li, N , Zhao, JY , Fei, P , Zhang, GM , Mao, JB & Rychwalski, PJ (2013) Findings of perinatal ocular examination performed on 3573, healthy full‐term newborns. Br J Ophthalmol, 97(5), 588–591. 23426739 10.1136/bjophthalmol-2012-302539 PMC3632968

[aos14586-bib-0014] Li LH , Wu WC , Li N , Lu J , Zhang GM , Zhao JY & Ma Y (2017): Full‐term neonatal ophthalmic screening in China: a review of 4‐year outcomes. Ophthalmic Surg Lasers Imaging Retina 48: 983–992. 29253301 10.3928/23258160-20171130-05

[aos14586-bib-0015] Loudot C , Fogliarini C , Baeteman C , Mancini J , Girard N & Denis D (2007): Rehabilitation on functional amblyopia in Morning Glory Syndrome. J Fr Ophtalmol 30: 998–1001. 18268439 10.1016/s0181-5512(07)79276-8

[aos14586-bib-0016] Ludwig CA , Callaway NF , Blumenkranz MS , Fredrick DR & Moshfeghi DM (2018): Validity of the red reflex exam in the Newborn Eye Screening Test Cohort. Ophthalmic Surg Lasers Imaging Retina 49: 103–110. 29443359 10.3928/23258160-20180129-04

[aos14586-bib-0017] Ma Y , Deng G , Ma J , Liu J , Li S & Lu H (2018): Universal ocular screening of 481 infants using wide‐field digital imaging system. BMC Ophthalmol 18: 283. 30376816 10.1186/s12886-018-0943-7 PMC6208088

[aos14586-bib-0018] Minotto I , Abdala N , Miachon AA , , Spinola e Castro M , Imamura P & Nogueira RG (2007): Basal encephalocele associated with morning glory syndrome: case report. Arq Neuropsiquiatr 65(4A): 988–991. 18094860 10.1590/s0004-282x2007000600013

[aos14586-bib-0019] Simkin SK , Misra SL , Battin M , McGhee CNJ & Dai S (2019): Prospective observational study of universal newborn eye screening in a hospital and community setting in New Zealand. BMJ Paediatr Open 3(1). 10.1136/bmjpo-2018-000376 PMC6361368 30815584

[aos14586-bib-0020] Sun M , Ma A , Li F , Cheng K , Zhang M , Yang H , Nie W & Zhao B (2016): Sensitivity and specificity of Red Reflex Test in Newborn Eye Screening. J Pediatr 179: 192–196.e194. 27640356 10.1016/j.jpeds.2016.08.048

[aos14586-bib-0021] Vinekar A , Govindaraj I , Jayadev C et al. (2015): Universal ocular screening of 1021 term infants using wide‐field digital imaging in a single public hospital in India ‐ a pilot study. Acta Ophthalmol 93: e372–e376. 25721891 10.1111/aos.12685

[aos14586-bib-0022] Wasilewski D , Zago RJ , Bardal AM et al. (2002): Importance of the ophthalmological evaluation in newborns. J Pediatr (Rio J) 78: 209–212. 14647776

[aos14586-bib-0023] Yamakiri K , Uemura A & Sakamoto T (2004): Retinal detachment caused by a slitlike break within the excavated disc in morning glory syndrome. Retina 24: 652–653. 15300100 10.1097/00006982-200408000-00032

